# Probiotic administration correlated with reduced diarrheal incidence and improved gut microbiota diversity in young goats

**DOI:** 10.3389/fvets.2025.1604638

**Published:** 2025-06-18

**Authors:** Mohamed Osman Abdalrahem Essa, Cheng Cheng, Jun Li, Xiao Han, Zhong Kang Wei, Layla Ahmed Mohammed Abdelhadi, Huda Ahmed Hassan, Saber Y. Adam, Hosameldeen Mohamed Husien, Ahmed A. Saleh, Darong Cheng

**Affiliations:** ^1^College of Veterinary Medicine, Yangzhou University, Yangzhou, China; ^2^College of Veterinary Medicine, Albutana University, Rufaa, Sudan; ^3^College of Animal Science and Technology, Yangzhou University, Yangzhou, China; ^4^Animal and Fish Production Department, Faculty of Agriculture (Al-Shatby), Alexandria University, Alexandria, Egypt; ^5^Jiangsu Co-innovation Center for Prevention and Control of Important Animal Infectious Diseases and Zoonoses, Yangzhou, China

**Keywords:** goat kids, probiotics, gut microbiota, diarrhea, *Enterococcus faecium*, *Bacteroides fragilis*, 16S rRNA sequencing

## Abstract

**Introduction:**

Probiotic interventions in young livestock are gaining attention for their potential health benefits.

**Methods:**

This study involved 15 weaned goat kids (2–3 months old; 10–15 kg body weight), including 10 healthy kids and 5 diarrheic kids. The kids were divided into three groups: Healthy Control (H, no treatment), Probiotic-Treated Healthy (T), and Diarrheic + Probiotic-Treated (D). All kids were maintained under standardized environmental conditions and fed a controlled diet (60% corn, 15% pea skin, 15% silage, 5% hay and 1% vitamin-mineral additives). Probiotic bacteria *Enterococcus faecium* and *Bacteroides fragilis* were administered via oral gavage at a concentration of (1 × 10^9^) CFU/mL for five consecutive days. Fecal samples were collected for sequencing of the bacterial 16S rRNA gene to analyze microbial composition.

**Results:**

Healthy groups exhibited significantly greater species richness and diversity compared to the diarrheal group (p < 0.01). The predominant phyla identified were *Pseudomonadota*, *Bacteroidetes*, and *Bacillota*.Increased levels of *Xylanibacter*, *UCG-055*, *Bacteroides*, and *Escherichia-Shigella* were noted in healthy treated kids, while *Prevotellaceae* UG_001 and *Proteus* decreased.

**Discussion:**

The findings highlight significant gut microbiota differences between healthy and diarrheal kids, suggesting that modifications in gut microbiota composition could alleviate diarrhea, contributing to preventive and therapeutic strategies for this condition.

## 1 Introduction

The term “gut microbiota” (GM) describes the varied population of microorganisms that live in animal intestines, including bacteria, fungus, viruses, and protozoa. These microbes are essential for immune system function, nutrient absorption, digestion, and general health. Numerous studies have demonstrated a clear correlation between the diversity and composition of bacteria in broiler chickens’ intestines and their development rate, feed conversion rate, and disease resistance (1–3). This intricate system is vital for both humans and animals, comprising a diverse array of microorganisms, including bacteria, viruses, fungi, and parasites (4, 5). Among these, the intestinal biota fulfills essential roles in digestion and overall health (6). Bacterial populations in the gut can be categorized as probiotics, transient visitors, or pathogenic, depending on their interactions with the host. Probiotics are particularly significant for maintaining the gut microbial environment, enhancing digestive function, and bolstering the immune response (7).

Animal intestines are intricate micro-ecosystems with a wide variety of microbiomes that are critical to the development of the host’s health. One of the rare species in Hainan, China, is the Hainan black goat, which is resistant and adaptable. Their gut biota is inextricably linked to these distinct physiological traits (8). GM plays a vital role in host physiology, health, and immune system maturation, enhancing intestinal self-recognition and immunological capacity, and it has the potential to mitigate diseases such as diarrhea and weakness. Despite extensive research on gut microbiome populations associated with diarrhea in various species, assessments specifically focusing on kids and their gut microbial communities have been inadequate until now (9–12).

Kids are particularly vulnerable to diarrhea, inflammation, and harmful microbial infections due to their still-developing immune systems at birth (13). Diarrhea in these kids is a prevalent symptom in animal husbandry, often associated with gastrointestinal dysfunction that impairs normal growth and can lead to increased mortality rates. Previous investigations have suggested that genetic variations within goat populations may influence the prevalence of diarrhea and contribute to fluctuations in health status (14, 15). Although a connection between changes in GM and diarrhea may exist, the specifics remain unclear. Moreover, while age-related factors potentially influencing intestinal microbiota have been suggested, they have not been deeply investigated. Pathogenic diarrhea is frequently linked to specific bacteria, yet the role of the overall intestinal biota is often overlooked (16, 17). Recent studies indicate that dysbiosis may be a primary contributing factor to diarrhea (18).

In this context, probiotics present a promising intervention for promoting the growth of beneficial organisms while inhibiting harmful microorganisms, thus preventing gastrointestinal infections. Probiotics influence the release of organic acids, digestive enzymes, and bioactive peptides, maintaining intestinal health and fostering beneficial bacteria with specific growth effects (19–21). Recent advancements have highlighted the potential of probiotics to improve gastrointestinal microbial composition.

and enhance both the immune system and overall health of young animals (22–24). The immune homeostasis regulated by oral administration of Lactobacillus and other probiotics was associated with decreased systemic inflammatory responses [reduction in C-reactive protein, Complement C3, and Immunoglobulin G (IgG) and the activated immunomodulation function of immune cells (25)].

*Bacteroides fragilis* ZY-312, a commensal anaerobic bacterium, has demonstrated probiotic properties, including enhancement of intestinal barrier integrity via upregulation of tight junction proteins (ZO-1, occludin) in previous studies (26–28). A class of bacteriocins produced by *E. coli* Nissle 1917 were also proven to be able to prevent intestinal inflammation and inhibit the competitive exclusion of *Enterobacteriaceae* (29). In addition, many strains of *Enterococcus* which inhabit human and animal guts are used as probiotics for humans, animals, and starter cultures in the food industry (30). *E. faecium* was proven to be an effective antibiotic alternative for the beneficial effects of enhancing animal health and growing performance (31). Moreover, *E. faecium* was also found to be able to improve the host intestinal epithelial defense program and limit the pathogenesis of enteric infection induced by *Salmonella enterica* and *C. difficile* (32). Kim et al. proved that the intestinal barrier function and pathogen tolerance which were improved by *E. faecium* were associated with the secretion of peptidoglycan hydrolase (SagA) (33).

This study investigates the probiotic potential of sheep-derived *Enterococcus faecium* and *B. fragilis* isolates in alleviating diarrhea in young goats. We hypothesize that these strains restore gut microbiota homeostasis, reduce intestinal inflammation, and enhance barrier function, thereby mitigating diarrhea in Jiangsu province’s goat population. This hypothesis is based on their exceptional qualities, which we suggest are influenced by both intestinal microbial interactions and the unique genetic factors of the kids in this region. However, understanding the relationship between the components of goat GM and diarrhea remains unclear. Thus, the primary aims of this study are to analyze the microbiota composition of fecal samples from kidscomprising healthy controls, healthy kids treated with probiotic bacteria, and diarrheal kids. Specifically, we aim to: (1) identify variations in bacterial communities between diarrheal kids and healthy controls, (2) determine the impact of probiotic treatment on GM diversity, and (3) explore the relationship between age and microbial composition in kids.

## 2 Materials and methods

### 2.1 Animals grouping and treatment protocol

In total, 15 weaned kids (10–15 kg.b.w) were randomly selected from kids goat farm located in the Lianyungang District of Guanyun City, Jiangsu Province. This group comprised 10 healthy kids, approximately 2 to 3 months old, and 5 kids exhibiting diarrhea. The selection criteria were as follows: Healthy kids were defined as those showing no clinical symptoms of illness, maintaining normal appetites, activity levels, and firm fecal consistency. Diarrheic kids were characterized by the presence of loose or watery stools lasting for at least two consecutive days, along with diminished activity or appetite (Supplementary Table S2). All kids were nearly 2 months old and weighed similarly, ranging between 10 and 15 kg. They were kept under uniform husbandry conditions, with constant access to clean water and feed, which consisted of a standard farm diet. Kids were maintained under controlled environmental conditions (ventilation, bedding) and fed a standardized diet (60% corn, 15% pea skin, 15% silage, 5% hay, 1% additives) (Table 1**)**.

**Table 1:** Composition of ingredients for goat feed.

Ingredient	%	Nutritional composition (per kg)
Corn	60	3.2 MJ ME, 8.5% crude protein
Pea skin	15	12% fiber, 2.5% fat
Silage	15	6.8% moisture, 1.8% calcium
Additives	1	Vitamins (A, D, E), trace minerals (Zn, Se)

Kids were housed in a controlled environment with adequate ventilation, bedding, and standard farm management practices. A male-to-female ratio of 1:4, representing 20% males and 80% females, was maintained consistently throughout the study. The farm is privately owned, and permission to collect fecal samples was granted for the period from October 10 to October 15, 2024. Kids were gradually weaned over 14 days by reducing milk replacer intake by 10% daily, transitioning to a solid diet (Table 1) by day 14. This minimized stress-related gut dysbiosis. The subjects were randomized into three groups:

- Healthy Control (H, *n* = 5): untreated healthy kids with no gavage administration.
- Probiotic-Treated Healthy (T, *n* = 5): healthy kids administered *Enterococcus faecium* and *Bacteroides fragilis*, which were gavaged with a probiotic combination.
- Diarrheic + Probiotic-Treated (D, *n* = 5): diarrheic kids receiving a gavage of a mixture of *E. faecium* and *B. fragilis* (MN334334) at a concentration of 1 × 10^9^ CFU/mL every 2 days.

The bacterial suspension was prepared at a concentration of 1 × 10^9^ CFU/mL with a 1:1 ratio of each species. On day 0, rectal swab samples were collected for baseline microbiota analysis.

Subsequently, the kids in the Healthy Group (T) and the Diarrhea Group (D) were gavaged with 5 mL of each probiotic bacterial suspension per 10 kg of body weight for five consecutive days. The control group (H) received no treatment. The dosage of the two bacterial mixtures was adjusted based on the weight of each kid to ensure proper administration (Table 2). The bacteria were cultivated under anaerobic conditions at 37°C for 48–72 h on Centers for Disease Control Anaerobic 5% Blood Agar (CDC; Cat# HB8511; Qingdao Hope Bio-Technology, Co., Ltd., China) or in fastidious anaerobe broth (FAB; Cat# LA4550; Solarbio, Inc., China) supplemented with 5% goat blood (Cat# TX0020; Solarbio, Inc., China) (34). Following plating on CDC containing 5% goat blood, colony-forming units (CFUs) were counted to determine bacterial concentration.

**Table 2:** Overview of sample groups and methodology.

Group	Group designation	Individuals	Sample replicate	Age (months)	Weight (kg)	Health status	Gavage treatment	Gavage dosage	Environmental conditions	Sample collection days	Sample codes
Healthy control group (H)	Healthy kids	5	3	2–3	10–15	No clinical signs of disease	No treatment	N/A	Controlled environment with bedding	Day 0, Day 3, Day 5	H0, H3, H5
Healthy group (T)	Healthy kids with Probiotics	5	3	2–3	10–15	No clinical signs of disease	Gavage with *Enterococcus faecium* and *Bacteroides fragilis*	10 mL/10 kg body weight	Controlled environment with bedding	Day 0, Day 3, Day 5	T0, T3, T5
Diarrhea group (D)	Diarrheic kids	5	3	2–3	10–15	Loose or watery stools, reduced activity	Gavage with *Enterococcus faecium* and *Bacteroides fragilis*	10 mL/10 kg body weight	Controlled environment with bedding	Day 0, Day 3, Day 5	D0, D3, D5

H: Healthy Control Group; T: Healthy Group (Probiotic Treatment); D: Diarrhea Group; N/A: Not Applicable; Age (Months): Age of the kids in months; Weight (kg): initial Body weight in kilograms; Gavage Treatment: Administration of treatments; Gavage Dosage: Dosage administered during gavage; Environmental Conditions: Description of the living conditions; Sample Collection Days: Days on which samples were collected; Sample Codes: Designation used for samples collected at different time points.

### 2.2 Sample collection

Three kids per group were selected for fecal sampling based on health status consistency (e.g., no clinical signs in controls, persistent diarrhea in the D group). Samples were collected on days 0, 3, and 5 using sterile swabs. Selection was based on ensuring representative individuals within each group, consistent with the inclusion criteria described in Section 2.1. The selection process was based on the following criteria:(1) Health Status: Only healthy lambs in the Healthy Control Group with no clinical signs of disease (as described in Section 2.1) were included in the selection for analysis. (2) Consistency Across Groups: The kids were chosen to ensure that they met the inclusion criteria specified for each group, with balanced representation from the Healthy Control Group, the Healthy *+ Enterococcus faecium and B. fragilis Group* (T0, T3, and T5), and the Diarrhea Group. (3) Non-Interference with Future Sample Collection: Once a kid was selected for a group, it remained part of that group for the duration of the study, ensuring consistency in the sampling process. Fresh fecal samples and rectal swabs were collected from three selected kids on day 0, day 3, and day 5 in replicate, respectively, using sterile cotton swabs (once a kid was selected, subsequent fecal and rectal swab collection could not be changed). The samples were numbered as follows: H0, H3, H5, T0, T3, T5, D0, D3, and D5. These samples were then stored in sterile plastic containers at −20°C and transported to the laboratory on ice within 2 h, subsequently frozen at −80°C for DNA extraction (Table 2). Briefly, to standardize comparisons, three kids per group were selected based on strict health criteria: healthy controls (no diarrhea, normal activity), diarrheic kids (watery stools >2 days), and treated kids (post-probiotic administration) (Supplementary Table S2). Fecal samples were collected at baseline and days 3 and 5 for 16S rRNA sequencing.

### 2.3 DNA extraction, PCR amplification of 16 SrRNA and sequencing

Genomic DNA isolated from fecal samples was performed according to the manufacturer’s instructions using the QIAamp Fast DNA Stool Mini Kit, as presented in Supplementary Table S1.

The PCR amplification conditions were set as follows: an initial denaturation step at 94°C for 3 min, followed by 24 cycles of 94°C for 5 s, 57°C for 90 s, and 72°C for 10 s, concluding with a final elongation step at 72°C for 5 min. To prepare indexed libraries for downstream next-generation sequencing (NGS) on the Illumina MiSeq platform, indexed adapters were concurrently appended to the ends of the 16S rDNA amplicons. Following the manufacturer’s recommendations, DNA libraries were multiplexed and loaded onto the Illumina MiSeq device (Illumina, San Diego, California, United States) using a 2 × 250 or 2 × 300 paired-end configuration for sequencing. The V3 and V4 sequences were processed, spliced, and analyzed by GENEWIZ (Guangzhou Genedenovo Biotechnology Co., Ltd., China).

### 2.4 Statistical analysis and bioinformatics

We employed the Trimmomatic software (v0.33) (35) and fastp software (v0.19.8) to correctly assign raw reads to their respective samples, utilizing each sample’s unique barcode. Quality filtering of the raw reads was conducted using cutadapt software (v1.9.1) (36) to identify and remove adapter sequences, thus ensuring high-quality target reads (37). Data were analyzed using QIIME2 (38), which included demultiplexing, merging, and *de novo* operational taxonomic unit (OTUs) selection from paired-end reads. We applied the DEBLUR algorithm (39), integrated within QIIME2, to align representative OTU sequences. Taxa classification into their respective OTUs was accomplished using a Naive Bayesian classifier trained on the Silva reference sequences (138 grouped at 99% similarity).

The Kruskal-Wallis test, a non-parametric method appropriate for non-normally distributed data, was selected to compare α-diversity indices (Chao1, Shannon) across groups. Beta diversity analysis used permutational multivariate analysis (PERMANOVA) with Weighted/Unweighted UniFrac distances, chosen to account for phylogenetic dissimilarity and community structure differences inherent to microbiome datasets (38, 40). To identify taxa with statistically significant differential abundances between groups, Linear Discriminant Analysis Effect Size (LEfSe) (41) was applied, as it combines non-parametric tests with biological effect size estimation. Statistical significance was determined at *p* < 0.05, with *p*-values adjusted for multiple comparisons using the Benjamini-Hochberg method (FDR < 0.05) to reduce false discovery rates in high-dimensional microbiome data. Results are reported as means. Principal Coordinates Analysis (PCoA) was conducted to visualize differences among fecal microorganisms. Rank and rarefaction curves were generated to assess the evenness, depth, and richness of the sequencing data.

Additionally, a power analysis was conducted using GPower 3.1 (https://gpower.software.informer.com/3.1) to determine the minimum sample size required to detect significant differences in microbial diversity (α = 0.05, power = 0.8, effect size = 1.2). The analysis indicated a minimum of 5 animals per group, aligning with our experimental design.

#### 2.4.1 Functional profile analysis of the intestinal bacterial community using phylogenetic investigation of communities by reconstruction of unobserved states

We revised our approach to the functional profiling of the intestinal bacterial community, utilizing phylogenetic investigation of communities by reconstruction of unobserved states (PICRUSt) software (v1.1.4) in conjunction with the Clusters of Orthologous Groups (COG) annotation databases. This methodology was employed to predict the Kyoto Encyclopedia of Genes and Genomes (KEGG) ortholog functions of the bacterial micro-population in kids (42). We examined changes in the functional dynamics of intestinal microorganisms through the Dunn test and analysis of variance (ANOVA).

## 3 Results

### 3.1 Analysis of sequencing data and taxonomy

**Figure 1:**
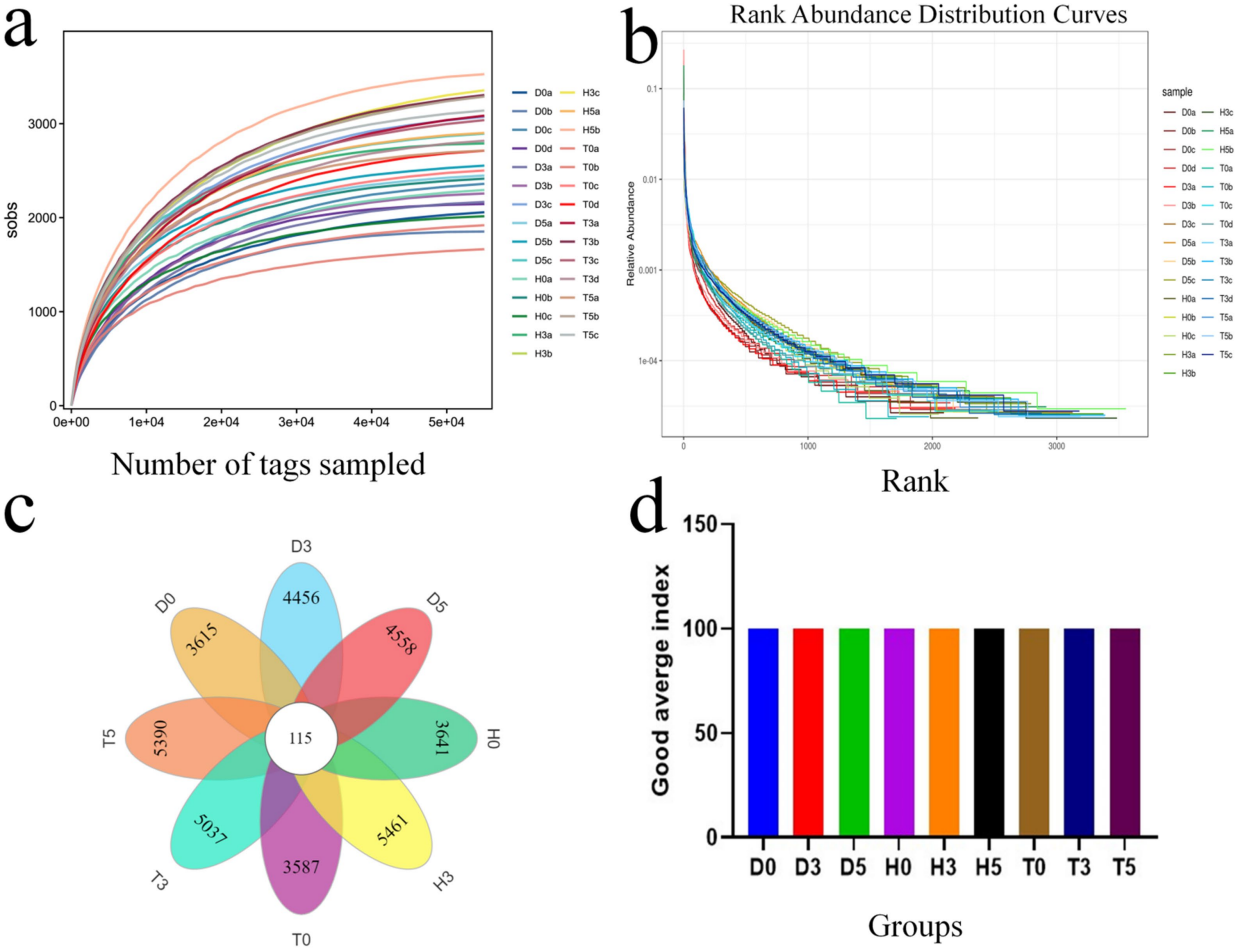
The species accumulation curve for each group; **(a)** Species accumulation curve for each group. **(b)** Rank abundance curve for each group. **(c)** Venn diagram analysis illustrating the overlap and feasibility of OTUs among the different groups. **(d)** Good average index.

An average of 33,989 paired-end reads per sample was produced, resulting in a total of 1,581,835 reads generated using the Illumina HiSeq 2,500 platform. After applying quality control protocols and excluding unqualified data, each sample yielded 2,477,419 high-quality reads, with an average of 21924.2 reads per sample (Table 3). As the sequencing depth increased, the slopes of the rarefaction curves gradually decreased, indicating a tendency toward saturation when the number of qualified sequences exceeded 40,000. This result demonstrates that the quantity and depth of sequencing were adequate for further analysis (Figure 1a). All fecal samples exhibited smooth rank abundances within 6,000, indicating a high degree of evenness (Figure 1b).

**Table 3:** Sample sequence information, D: diarrhea group, H: control group T: healthy treatment with bacteria group.

Groups	Raw reads	Clean reads	OTU	Merged
D0	37,549	375,339	3,615	368,951
D3	30,568	305,592	4,456	300,687
D5	29,945	209,309	4,558	29,455
H0	317,132	316,989	3,641	311,353
H3	331,985	253,762	5,461	325,980
H5	20,239	202,319	1,323	198,901
T0	429,582	429,392	3,587	422,117
T3	352,136	352,030	5,037	346,404
T5	32,699	32,687	5,390	321,249

H: Healthy Control Group; T: Healthy Group (Probiotic Treatment); D: Diarrhea Group; 0: Day 0 (Baseline); 3: Day 3 (Post-Treatment); 5: Day 5 (Post-Treatment).

According to the DEBLUR program, a total of 3,706 OTUs were assigned from all samples, with a range of 67 to 210. Samples from groups H, D, and T yielded 264.4, 258.6, and 311.2 OTUs, respectively, based on 97% species similarity (Table 3). Notably, 115 of these OTUs were present in every group, designating them as core OTUs (Figure 1c). Approximately 73.6% of all OTUs were categorized as core OTUs. Figure 1d shows the good average index.

### 3.2 Microbial diversity index in different groups

**Figure 2:**
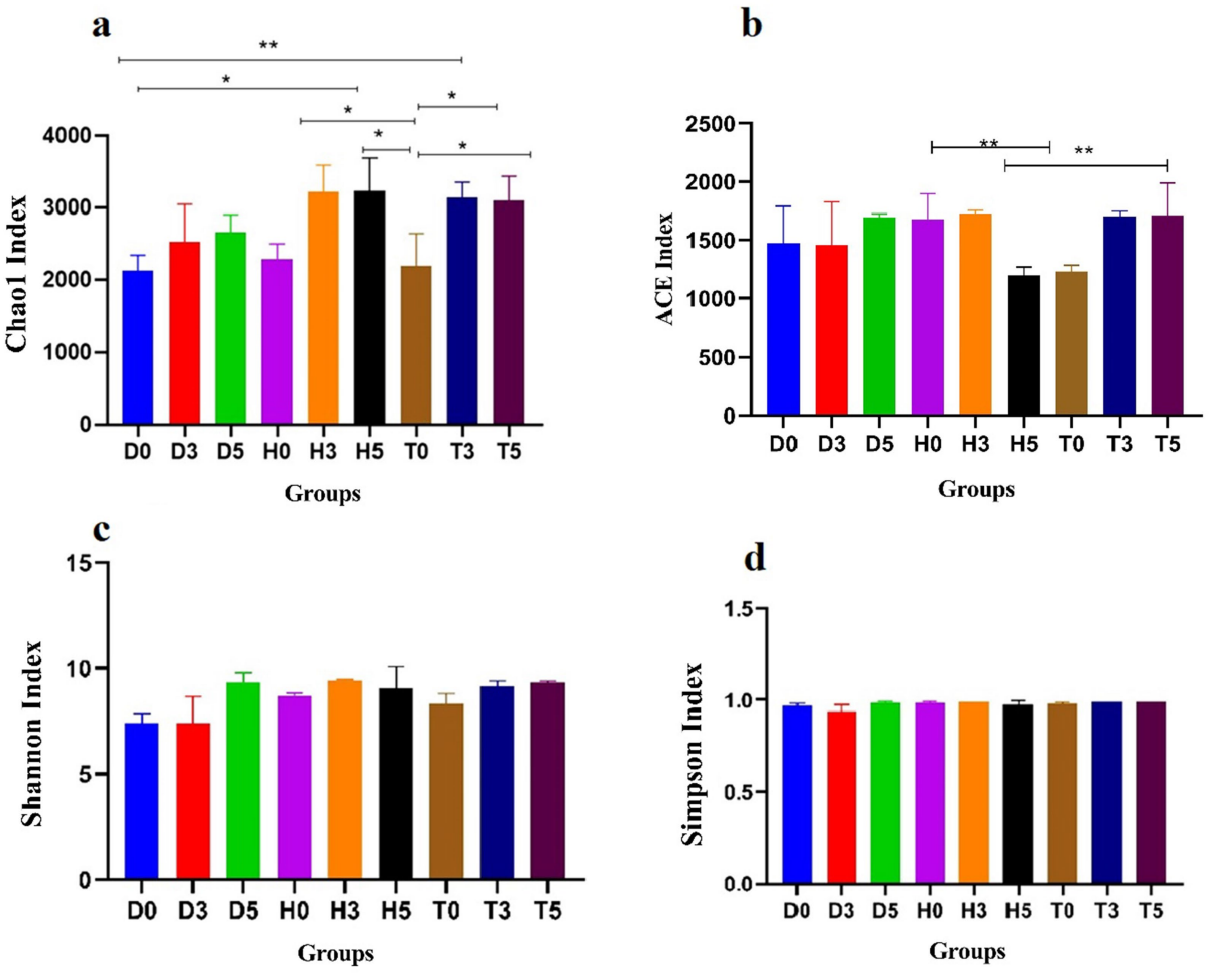
Comparative analysis of gut microbial diversity among the H, T, and D groups. Panels **(a–d)** display the Chao, ACE, Shannon, Simpson, and Good’s average indices. H represents the healthy control group, T denotes the healthy treatment group with bacteria, and D indicates the diarrhea group. Data are presented as mean ± standard deviation (*p < 0.05, **p < 0.01).

The microbial community’s diversity and abundance were assessed using various α-diversity indices. The Abundance based Coverage Estimator (ACE) estimator revealed average OTUs counts of 24,056, 22,996, and 55,328 for groups D, H, and T, respectively, indicating differences in microbial abundance. Similarly, the Chao1 estimator provided comparable average OTUs counts for the samples in these groups (Figures 2a,b). Diversity indices (Shannon, Simpson) revealed significantly higher microbial diversity in treated groups compared to diarrheic and control groups (*p* < 0.01, Figures 2c,d). Furthermore, good’s coverage estimates for groups D, H, and T were 100% for each group, indicating excellent coverage of the microbial community (Figure 2d).

**Figure 3:**
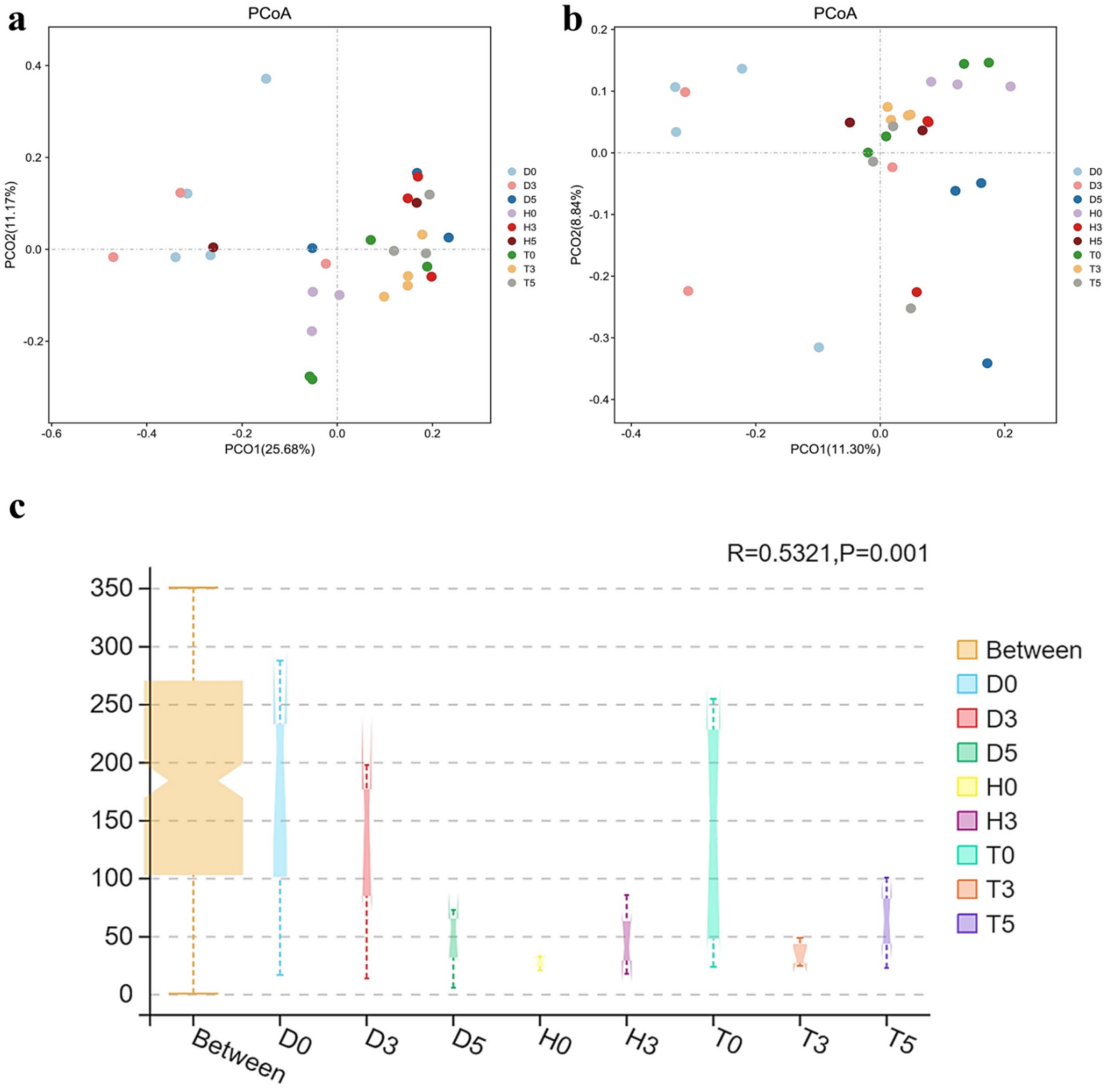
**(a)** PCoA derived from the Unweighted UniFrac distance matrix. **(b)** PCoA derived from the Weighted UniFrac distance matrix. Each sample is represented by a point on the map, illustrating the differences in gut microbiota between groups, expressed as the distance between them. **(c)** ANOSIM analysis: “Between” represents the difference between the groups; the closer the R-value is to 1.

The variations between individual samples or groups were clearly illustrated by the PCoA of the UniFrac distance matrix. The microbiota from group D0 visually separated from the other two subgroups, D3 and D5, clustering along the primary coordinates 1 and 2 (Figure 3a). Moreover, samples within each group tended to cluster together, with the exception of D0, D3, D5, and T5, suggesting minimal differences in community structure within groups. However, D0 and D5 exhibited distinct microbial compositions (Figure 3b). Additionally, the ANOSIM analysis indicated that the differences between groups were greater than those observed within samples (*R* = 0.5321, *p* < 0.001) (Figure 3c).

### 3.3 Differences in gut microbiota composition among the three groups

**Figure 4:**
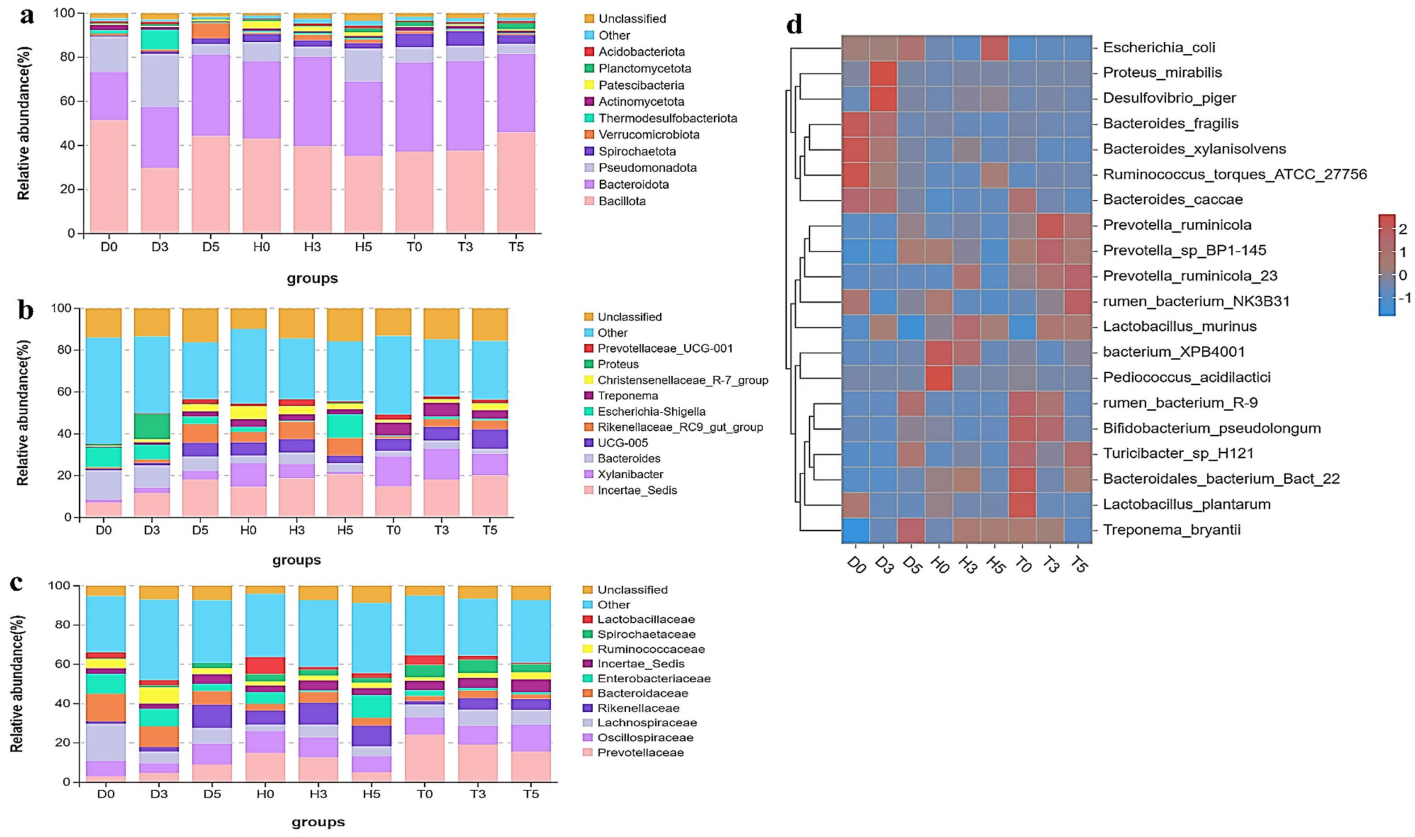
Microbial composition of different samples. Each bar represents the average relative abundance of bacterial taxa among groups. **(a)** Taxa assignment at the phylum level. **(b)** Taxa assignment at the family level. **(c)** Taxa assignment at the genus level. **(d)** Hierarchically clustered heatmap of taxonomic analysis at the genus level for each group.

This study explored the arrangement and composition of the gut bacterial community at various taxonomic levels. Analysis of phylum assignments revealed a total of 12 phyla, with *Bacillota* (51.46, 36.96%) being the most prevalent phylum across the 29 samples, followed by *Bacteroidota*. The phylum *Pseudomonadota* was detected in high abundances in samples D3b, D0b, D0d, and D0a (23.94, 22.51, 21.81, and 15.16%, respectively), while *Spirochaetota* and *Patescibacteria* (7.32, 7.09, 5.07, 2.83, 2.07, and 2.40%) were found in samples H0a, H0b, H3a, H3c, H0c, and H5b. Notably, only the T0d and T5c samples exhibited *Planctomycetota*, which were identified as decreasing and increasing in the H5a, D3c, H0a, and H3a samples (Figure 4a). Other phyla, including *Actinomycetota*, *Verrucomicrobiota*, *Thermodesulfobacteriota*, and *Acidobacteriota*, were represented with lower abundances. In addition to phylum-level analysis, bacterial abundances were examined at various taxonomic units, including families and genera (Figure 4).

At the family level, 10 families were identified, including Lactobacillaceae, Spirochaetaceae, Ruminococcaceae, Incertae Sedis, Enterobacteriaceae, Bacteroidaceae, Rikenellaceae, Lachnospiraceae, Oscillospiraceae, and Prevotellaceae (Figure 4b). The families Prevotellaceae and Oscillospiraceae were classified as core families present in all groups; the most dominant family was Lachnospiraceae (18.83 and 7.99% in D0 and D5), while Lactobacillaceae and Spirochaetaceae were absent in both D0 and D5. The healthy control and health treatment groups exhibited the highest abundance of Prevotellaceae, with T0 (24.20%), T3 (19.20%), T5 (15.5%), H0 (14.88%), and H3 (12.67%), followed by Oscillospiraceae (13.67, 11.03, 10.17, 9.54, 8.83, and 8.22% in T5, H0, H3, T3, T0, and H5, respectively).

To further evaluate the changes in bacterial composition at the genus level during diarrhea, a total of 10 genera were identified (Figure 4c). The genus Incertae Sedis was predominant in diarrheic samples and *Bacteroides* (D0 vs. D3: 14.09 and 10.51%) were noteworthy, along with *Proteus* (12.03% in D3). *Escherichia-Shigella* abundance was higher in healthy controls (H5: 11.28%) and diarrheic groups (D0: 9.48%, D3: 7.05%) compared to probiotic-treated groups. While *Bacteroides* (6.90–14.09%) was more abundant in the diarrheal groups compared to the other two groups, *Escherichia-Shigella* (0.41–1.16%) showed lower abundances in the treatment groups than in the diarrhea and healthy control groups. In terms of *Xylanibacter*, the health treatment and healthy control groups were more dominant than the diarrheal groups, ranging from 14.74 to 4.05%. Interestingly, seven of these genera were listed among the top 20 abundant taxa (Figure 4d). According to these findings, GM composition of healthy treatment and diarrheal kids differed significantly.

**Figure 5:**
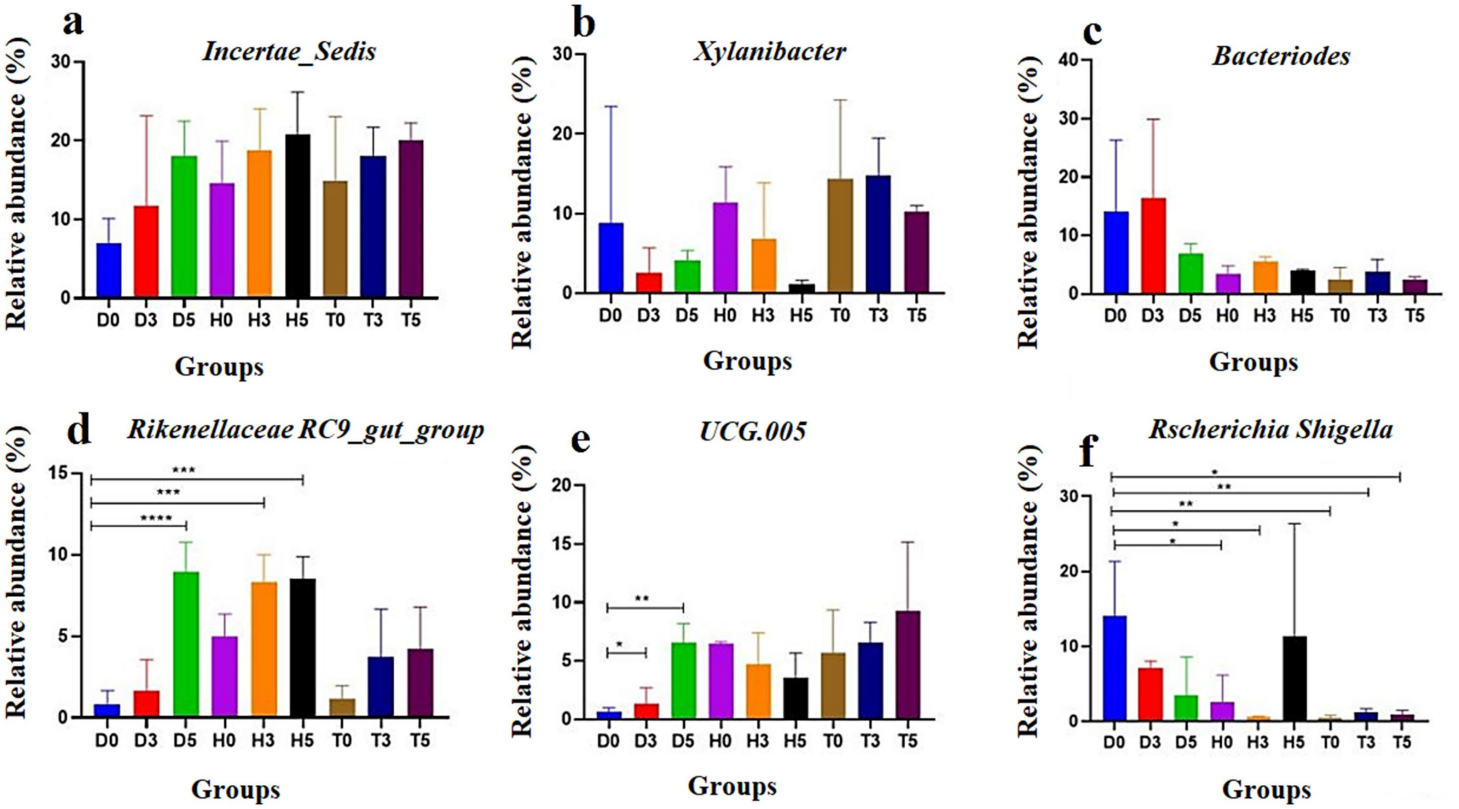
The abundance of the top six bacterial genera in goat kid fecal microbiota for each group: **(a)** *Incertae Sedis*, **(b)** *Xylanibacter*, **(c)** *Bacteroides*, **(d)** *UCG-005*, **(e)** *Rikenellaceae RC9* gut group, and **(f)** *Escherichia-Shigella*. Data are presented as the mean and standard deviation (SD); *p < 0.05, **p < 0.01, ***p < 0.001, and ****p < 0.0001.

In addition, the heatmap showed the distribution of bacterial species throughout each sample. The results indicate significant changes in GM of both the diarrheal and healthy treatment groups compared to healthy controls. As illustrated in Figures 5a–f, a total of 20 and 7 bacterial taxa were abundant in the control and health treatment groups compared to the diarrheal groups, respectively. At the genus level, *Incertae Sedis*, *Bacteroides*, *UCG.005*, *Rikenellaceae RC9 gut group*, and *Escherichia-Shigella* were significantly enriched in the diarrheal group, while *Xylanibacter*, *Incertae Sedis*, and *UCG-005* were predominantly associated with the healthy group based on the heatmap analysis.

### 3.4 LEfSe analysis and T-test analysis

To ensure comprehensive detection of the taxonomic composition, LEfSe analysis, in conjunction with cladogram scores, was employed to identify the taxonomic compositions at the family, order, genus, and species levels among the groups. The analysis revealed that the relative abundances of *Lachnospiraceae* and *Lachnospirales* were predominant in the diarrheal group (D0), while the most dominant families in the D3 group were *Micrococcaceae* and *Conobacteriaceae*. Additionally, when comparing D3 to D5, the most dominant bacteria identified were *Proteus*, *Clostridia UCG* (genus), and *Lactobacillus johosnii*. In the control group comparison of H0 vs. H3, the most prevalent bacteria included *Sphingomonadales*, *Sphingomonadaceae*, *Klebsiella*, and *Pediococcus acidilactici*. Furthermore, in the comparison between the healthy treatment group and control groups (H3 vs. T0), the results indicated that *Bacteroidales*, *Rikenellaceae*, *Alistipes*, and *Bacteroides caccae* exhibited higher abundances. Lastly, among the health treatment groups, the most abundant bacteria identified were *Bacillota*, *Clostridia*, and *Christensenellales* in the *Christensenellaceae* family.

### 3.5 Correlation of gut microbiota with healthy control, health treatment levels, and diarrhea across days

**Figure 6:**
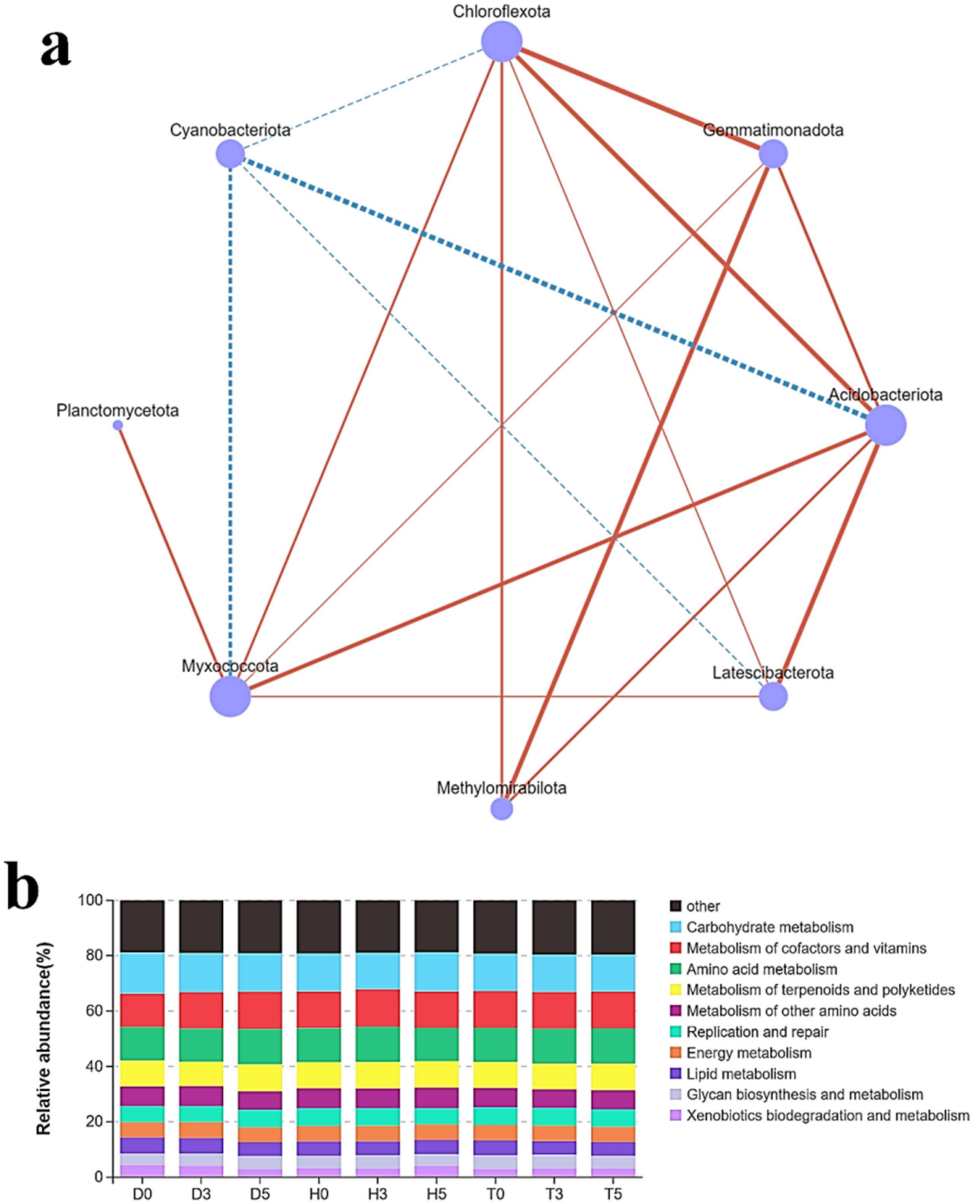
Pearson correlation analysis reveals relationships between various microbes. Red lines indicate positive correlations, while blue lines represent negative correlations. D: diarrheal group, H: control group, T: healthy treatment with probiotic bacteria **(a)**. The PICRUST analysis annotates the relative abundance among different groups of goat kids **(b)**.

APICRSt2 metagenomic functional prediction was conducted to link the microbial genera to the KEGG metabolic database. This analysis aimed to evaluate the functional capacities of mucosal taxa across the three groups. A total of 30 metabolic pathways exhibited significant differences among the groups (Figure 6). The diarrheal group (D5) displayed significantly enriched pathways, particularly those related to the biosynthesis of secondary metabolites and lipid metabolism, which indicate adaptation and microbiome recovery. In contrast, the D3 and D0 groups exhibited reduced pathways associated with amino acid, nucleotide metabolism, and cell motility. The control group (H0) showed substantial increases in pathways related to carbohydrate and energy metabolism (H3 and H5), as well as in membrane transport, signal transduction, and biosynthetic pathways on the healthy treatment side. While T0 was similar to H0, T3 demonstrated an increase in carbohydrate metabolism and lipid metabolism compared to T0, reflecting the recovery of the microbiome. Additionally, T5 showed enhancements in energy metabolism, glycan biosynthesis, and membrane transport.

### 3.6 Pearson correlation analysis

There was a significant correlation between the health recovery of young kids and the gut probiotic bacteria at the phylum level of the microbiota (Figure 6a). A negative correlation was found with the relative abundance of *Actinobacteriota*. For *Chloroflexota*, the relationship with relative abundance was significant at *p* < 0.05, while for *Acidobacteriota*, it reached significance at *p* < 0.05. Additionally, a positive correlation was observed between the relative abundance of *Chloroflexota* and the stomach mucosa, as well as with *Myxococcota* (*p* < 0.001). Gut methanogen activity positively correlated with the relative abundances of *Acidobacteriota*, *Gemmatimonadota*, and *Chloroflexota* (*p* < 0.05). Furthermore, the activity of *Latescibacterota*, which includes *Chloroflexota*, *Methilomirobilota*, and *Myxococcota*, was positively correlated with their relative abundances (*p* < 0.001). Interestingly, no correlation was found with *Cyanobacteria* (*p* < 0.05).

## 4 Discussion

Recent microbiome studies have illuminated the pivotal role that gut microbiota (GM) play in host health, highlighting how alterations in GM can directly influence an individual’s overall health and susceptibility to various diseases (43). While a significant amount of research has focused on the changes in gut microbiomes in animals before and after illness, there is limited information regarding the colonization process of GM in goat kids, particularly during their weaning period. This study aimed to investigate the fecal microbiota composition and its alterations throughout this developmental stage in goat kids, revealing that the gut microbial ecosystem in these animals develops through distinct, consecutive stages over time.

Previous research has identified variations in mammalian intestinal microbiota associated with factors such as metabolism, physiology, and immunology (44). However, studies focusing on the GM of kids at different ages and health statuses remain scarce. Our analysis of the diversity and abundance of bacteria in the rectal contents of goat kids across varying health conditions indicated significant differences in microbial richness and diversity among the three studied groups diarrheic (D), healthy (H), and treatment (T) with the T group showing the highest abundance of operational taxonomic units (OTUs), indicative of a more diverse GM. The Shannon-Wiener diversity indices supported these findings, showing that the T group exhibited the highest diversity (97.88), while the Simpson index indicated lower diversity in the H group. Robust coverage across all groups confirmed distinct microbiota compositions, revealing spatial relationships and clustering among microbial communities, particularly within the D group, which exhibited distinct subgroups and minimal intragroup variation. This emphasizes the importance of spatial relationships in understanding microbial dynamics.

Our observations align with those of Zhang et al. (45), who noted that diarrheic goat kids displayed significant differences in terms of diversity and abundance compared to the other groups. *Bacteroides* were the predominant bacteria in the guts of diarrheic kids, accompanied by increased levels of *Paeniclostridium* and *Clostridium*_*sensu_stricto*_1, while the percentages of *Rikenellaceae_RC9*_gut_group, *Ruminococcaceae*_*UCG-005*, and *Christensenellaceae_R-7*_group declined sharply. Conversely, the recovery group showed a notable increase in *Xylanibacter,* a genus linked to gut homeostasis.

Our investigation across various taxonomic levels provided fundamental insights into the structure and composition of the gut microbiota in different treatment and health groups. At the phylum level, *Bacillota* and *Bacteroidota* predominated all sampled groups, emphasizing their essential roles in gut ecology. Specific samples (D3b, D0b, D0d, and D0a) predominantly featured these phyla, reinforcing earlier research that recognizes the critical ecological functions of ruminant gut microbes. Notably, previous research on Hainan black kids reported *Firmicutes, Bacteroidota,* and *Pseudomonadota* as prevalent phyla; however, our findings revealed higher levels of Bacillota in smaller kids (except in the control group), paralleling results observed in sheep (46, 47). It has been suggested that Bacteroides may aid in enhancing nutrient absorption in the host (45), with the concentration of *Bacteroidetes* correlating with the cellulose and polysaccharides ingested by the host (48). In contrast, *Pseudomonadota* is crucial for assessing intestinal health and upholding the structural stability and balance of GM (49). Furthermore, rare phyla such as *Planctomycetota*, *Patescibacteria*, and *Spirochaetota* exhibited sample-specific occurrences, reflecting unique microbial habitats and functional contributions within the gut.

The identification of ten bacterial families including *Prevotellaceae*, *Oscillospiraceae*, and *Lachnospiraceae* highlights the diversity and specificity of bacterial populations. Notably, *Oscillospiraceae* and *Prevotellaceae* emerged as core families present in all groups, underscoring their critical roles in maintaining intestinal homeostasis. The prominent presence of *Lachnospiraceae* in groups D0 and D5, alongside the absence of *Lactobacillaceae* and *Spirochaetaceae*, raises concerns about potential dysbiotic conditions or treatment-related variations. Furthermore, the notable prevalence of *Prevotellaceae* in both healthy treatment and control groups corroborates previous studies linking this family to anti-inflammatory effects and balanced GM (50).

At the genus level, significant differences were noted among the diarrheal, healthy control, and treatment groups. Diarrheal samples were enriched with genera such as Bacteroides, *Proteus*, *Escherichia-Shigella*, and *Incertae Sedis*, which are associated with gut dysbiosis and disease. *Escherichia-Shigella,* recognized for its pathogenic potential, exhibited a marked decrease in treatment groups compared to diarrheal and control groups, highlighting treatment efficacy in reducing its abundance (51). In agreement, De Filippo et al. (50) noted consistent results across a broader body of research indicating that probiotics and targeted therapies can reduce harmful bacteria and enhance microbial diversity, promoting gut health and disease resistance. Additionally, Zhang et al. (45) documented a widespread presence of *Bacteroides* in diarrheal children, pointing to its role as a typical gut microbe in ruminants that may cause endogenous infections when host immunity or GM is compromised. In contrast, a study of adult yaks with diarrhea revealed an increased relative abundance of *Proteobacteria* alongside a significant decrease in *Bacteroidetes* (52). The changes in GM composition may reflect an overall reduction in inflammation and a restoration of beneficial microbial communities. Positive modulation of these taxa suggests a connection to their ecological niches and interactions within the gut. Liu et al. (53) similarly noted a significant overrepresentation of *Escherichia coli* in adult yaks and calves experiencing diarrhea. The observed increase in diversity among gut microorganisms in the diarrheal group may link to gastrointestinal illnesses, inflammation, and related health concerns. Effective treatments tend to promote beneficial or commensal species while diminishing harmful bacteria like *Escherichia-Shigella,* underscoring the importance of targeted therapies in alleviating dysbiosis-related diseases.

The studies conducted by various authors highlight the significance of gut microbiota diversity regulation through different strategies, including dietary supplementation, to improve the health and performance of livestock such as kids and pigs. Kong et al. (52) found that the dietary addition of tea polyphenols (4 g/kg) in weaned goat kids effectively maintained gut microbiota homeostasis, enhanced antioxidant and immune functions, and reduced inflammation, with a significant increase in beneficial microbiota such as *Verrucomicrobiota, Candidatus Soleaferrea*, and *Prevotella*. Furthermore, there was an increase in the Simpson index of diversity, and the supplementation activated intestinal defense mechanisms through the modulation of the TLR4/MyD88/NFκB signaling pathway. In exploring the dynamics of gut microbiota, Zhang et al. (45) revealed that healthy kids exhibited higher species richness and diversity compared to diarrheic kids, identifying *Firmicutes* and *Bacteroidetes* as the dominant phyla, with notable shifts in populations such as Bacteroides and *Clostridium sensu stricto 1* in diarrheic cases. Cheng et al. (34) investigated *B. fragilis*, derived from sheep, demonstrating a survival rate of 38.89% in gastric simulation and a 92.22% survival rate in intestinal fluids, effectively alleviating diarrhea in 80% of treated lambs and restoring gut microbiota diversity by reducing pathogens like *Aerococcus suis* and *Corynebacterium camporealensis*. Additionally, Yao et al. (54) isolated two strains of *Enterococcus faecium* (DC-K7 and DC-K9), which significantly enhanced the abundance of beneficial microbes and decreased harmful microbes in conditions of antibiotic-induced dysbiosis, indicating their potential for restoring gut microbiota balance. Quilcate et al. (55) established that breed-specific gut microbiota significantly correlated with meat quality traits in cattle, where *Christensenellaceae* R-7 and *Alistipes* were positively associated with marbling and muscle area, underscoring the microbiota’s role in livestock productivity. Lastly, Li et al. (56) demonstrated that glycerol monolaurate complex (GML) improved the reproductive performance of sows by shortening delivery intervals and reducing TNF-α levels in both sows and piglets, while the 0.2% GML supplementation positively influenced microbial diversity in piglets, indicating its potential as a safer alternative to antibiotics. Together, these studies emphasize that probiotic manipulation, dietary interventions, and careful management of gut microbiota are crucial for enhancing health and productivity in livestock while mitigating the adverse effects associated with antibiotic use.

By linking microbial taxa to KEGG metabolic pathways through PICRUSt2 metagenomic functional predictions, we gained valuable insights into the functional potential of GM across the three groups. Variations in microbial activity and adaptations to diarrheal, healthy control, and treatment conditions were reflected in the identification of critical metabolic pathways enriched differently across these circumstances. Particularly notable was the enrichment of pathways associated with lipid metabolism and secondary metabolite biosynthesis in the diarrheal group (D5). These KEGG pathways are fundamental to the GM’s recovery process amidst disease control and infectious disease responses (57). Prior studies on antibiotic usage in mice during infancy indicate that pulsed antibiotic therapy significantly impacts the structural diversity and dynamics of gut bacteria, often delaying microbiota recuperation (58). Such pathways likely signify microbial adaptability and the gut microbiome’s potential recovery responses to illness.

LEfSe analysis and linear discriminant analysis (LDA) scores were employed to identify bacterial taxa across various taxonomic levels in therapeutic contexts, providing a comprehensive understanding of GM composition. Dysbiotic states, such as diarrhea-related dysbiosis, can result in microbial imbalances and inflammation (57). In our study, *Lachnospiraceae* and *Lachnospirales* were linked to dysbiotic states, whereas the D3 group appeared to shift toward microbial recovery. Notably, the presence of *Pediococcus acidilactici*, a stable and beneficial microorganism, was observed in healthy individuals (58). These findings suggest that environmental or pharmacological interventions may have modified microbial ecology. According to LEfSe data, the case group exhibited significantly higher levels of genera including *Alistipes, Solibacillus, Bacillus,* and *Prevotellaceae_UCG_003,* with four of these among the top 20 most abundant taxa. Recent findings resonate with our results, highlighting the relevance of these bacterial species in the context of yak diarrhea.

Briefly, the present findings highlight the gut microbiome’s critical role in diarrheal health outcomes in goat kids. Key genera differing between groups included *Xylanibacter* (enriched in healthy kids) and *Escherichia-Shigella* (reduced post-treatment), while dominant phyla such as *Bacteroidetes* (associated with fiber digestion) and *Bacillota* (linked to gut barrier function) underscored functional resilience. Genus-level shifts, including increased *Xylanibacter* (health-associated) and decreased *Escherichia-Shigella* (pathogen-linked), suggest probiotic efficacy in restoring microbial balance. Core families like *Lachnospiraceae* (gut homeostasis) and *Prevotellaceae* (anti-inflammatory) were altered by treatment, whereas less abundant phyla (e.g., *Actinomycetota*) showed no significant trends. These compositional changes identify potential biomarkers for gut health and therapeutic targets.

Lastly, this study presents insightful findings regarding the potential of probiotics to influence gut microbiota and alleviate diarrheal symptoms in goat kids, highlighting *Xylanibacter* and *Escherichia-Shigella* as key taxa associated with intestinal health. However, a few limitations should be noted. The small sample size (*n* = 5 per group, with only 3 sequenced per time point) may limit statistical power, potentially overlooking subtle microbial dynamics. Additionally, fecal samples were used instead of samples obtained directly from the intestines, which could limit our understanding of the gut microbiome in diarrheal kids. This study did not perform strain-level genomic characterization or comprehensive safety profiling (e.g., virulence factors, antibiotic resistance genes) of the administered probiotics. While preliminary survival assays indicated robustness in gastric conditions, future work must validate strain safety and genetic stability to ensure suitability for livestock use. Nonetheless, fecal samples remain valuable for examining diseases in both humans and animals, providing reliable information regarding the host (59). Furthermore, it is important to consider that individual variations may dominate differences in gut microbiome composition, regardless of collection-processing techniques or sampling dates (60). Additionally, while 16S rRNA gene sequencing is effective for elucidating microbial community relationships with disease, establishing cause-and-effect connections remains challenging due to limitations in taxonomic assignments and reference databases (60, 61). The lack of clinical scoring or functional markers (e.g., inflammation assays) also constrains the ability to directly link microbial changes to host health outcomes. Future research should focus on larger cohorts, standardized clinical metrics, and multi-omics approaches (metagenomics and metabolomics) to further validate these findings, uncover underlying mechanisms, and enhance probiotic formulations for effective herd management. Integrating these methodologies in future studies will help elucidate host–microbe interactions and improve herd management strategies.

## 5 Conclusion

The present study highlights the important role of probiotics in promoting gut health and alleviating the adverse effects of diarrhea in young kids. The observed fluctuations in microbial richness and diversity following probiotic treatment suggest potential avenues for both preventative and therapeutic strategies in livestock management. The identification of specific bacterial taxa, such as *Xylanibacter*, *UCG-055*, *Bacteroides*, *Rikenellaceae_RC9_gut_group*, and *Escherichia-Shigella*, provides valuable indicators of diarrhea, aiding in early detection and intervention by clinicians and farmers. However, this study has notable limitations. Future research should aim to expand the sample size and include a more diverse cohort to better understand the variability in microbial responses. Additionally, it is essential to establish standardized guidelines for assessing intestinal health based on our findings, which could provide a foundational framework for ongoing research and practical applications. Incorporating microbial profiling, inflammation biomarkers, nutritional assessments, clinical observations, and probiotic efficacy into routine evaluations will enable stakeholders to make informed decisions that enhance animal health and welfare. Further research is crucial to deepen our understanding of the complex interactions within the gut microbiome and to clarify the mechanisms by which probiotics exert their beneficial effects. Investigating the influences of diet, environmental factors, and genetic variability on gut health will enrich our knowledge and facilitate more tailored interventions.

## Data Availability

The original contributions presented in the study are included in the article/Supplementary material, further inquiries can be directed to the corresponding author.
