# Supplementary material for: Probiotic administration correlated with reduced diarrheal incidence and improved gut microbiota diversity in young goats
Source: Front Vet Sci. 2025 Jun 18;12:1604638. doi: 10.3389/fvets.2025.1604638 (PMC12213349; doi:10.3389/fvets.2025.1604638)
Supplement: Supplementary file 1 [file Data_Sheet_1.pdf]

Supplementary File (1); Supplementary Tables

Table S1. Workflow for DNA Extraction and Sequencing of Fecal Samples

Steps	Protocol	Reagent	Manufacturer	Notes
**Fecal Samples**	Fresh fecal sample collected in sterile tubes	-	-	Sorted at -80°C until DNA extraction
**DNA Extraction**	Mechanical and Chemical	QIAamp Fast DNA Stool Mini Kit	Qiagen, Cat No. HB8511	Used per manufacturer's protocol
**DNA Quality and Quantity Test**	Nanodrop spectrophotometry and agarose gel	Agarose gel (1%)	Thermo Fisher; Sigma Aldrich	A260/A280 ratio and gel visualization
**PCR Amplification and Primer**	V3-V4 region of 16S rRNA	314F: CCTACGGGNGGCWGCAG<br>806R: GGACTACHVGGGTATCTAAT<br>Tag polymerase	Thermo Fisher	Standard PCR conditions applied
**Amplicon Purification**	Magnetic bead purification	AMPure	-	1.8:1 bead-to-sample ratio
**Library Preparation**	Indexing and adapter ligation	Next-generation sequencing	Illumina MiSeq platform	Following Illumina protocol

Supplementary File (1); Supplementary Tables

Table S2. Summary of Clinical Observations in Different Treatment Groups Over Time.

Group	Day 0	Day 3	Day 5	Clinical status
**Control(H)**	Normal	Normal	Normal	Noabnormal signs observrd
**Health probiotic (T)**	Lose stool	Slightly improved	Normal	Improvement noted
**Diarrhea(D)**	Diarrhea	Slightly improved	Normal	Recovery observed by Day5
